# p38 mitogen-activated protein kinase–induced nuclear factor kappa-light-chain-enhancer of activated B cell activity is required for neuroprotection in retinal ischemia/reperfusion injury

**Published:** 2012-07-26

**Authors:** Shao-Yun Jiang, Yuan-Yuan Zou, Jian-Tao Wang

**Affiliations:** 1 School of Dentistry, Tianjin Medical University, Tianjin, China; 2 Eye Center, Tianjin Medical University, Tianjin, China; 3 Dohney Eye Institute, Keck School of Medicine, University of Southern California, Los Angeles, CA

## Abstract

**Purpose:**

In our previous study, nuclear factor kappa-light-chain-enhancer of activated B cells (NF-κB) played a neuroprotective role in retinal ischemia/reperfusion (I/R) injury in rats. However, the mechanism of NF-κB neuroprotection is still unclear. We hypothesize that p38 mitogen-activated protein kinase (MAPK) is expressed and NF-κB activity induced by p38 MAPK plays a neuroprotective role through antiapoptotic genes (B-cell lymphoma [*Bcl*]*-2* and *Bcl-XL*) in retinal cells in retinal I/R injury.

**Methods:**

Retinal ischemia was induced by elevating intraocular pressure in rats. After retinal I/R, the *p38 MAPK*, *NF-κB p65*, *Bcl-2*, and *Bcl-XL* mRNA levels were measured with real-time polymerase chain reaction. NF-κB p65 activity was assessed with NF-κB enzyme-linked immunosorbent assay in retinal I/R injury and after application of the p38 MAPK inhibitor (SB203580). Furthermore, SB203580 and *NF-κB p65* short interfering RNA (siRNA) were used in retinal I/R injury to examine the effects on Bcl-2 and Bcl-XL levels and nucleosome release in the retina and cell survival in the ganglion cell layer.

**Results:**

The mRNA levels of *NF-κB p65* and *p38 MAPK* reached a peak at 6 h after retinal I/R and then decreased gradually. The mRNA levels of *Bcl-2* and *Bcl-XL* significantly increased at 2, 4, and 6 h, peaked at 8 h, and decreased gradually, but remained at a higher level compared with the normal control, which was accompanied by an increase in NF-κB p65 in nuclear extracts. After application of SB203580, the increase in the NF-κB p65 levels in the nucleus induced with I/R was completely abolished, and the mRNA expression of *Bcl-2* and *Bcl-XL* decreased significantly compared with the I/R controls. In addition, *NF-κB p65* siRNA inhibited Bcl-2 and Bcl-XL expression. Inhibition of the p38 MAPK-NF-κB pathway (using SB203580 or *NF-κB p65* siRNA) increased retinal nucleosome release and decreased the number of ganglion cells.

**Conclusions:**

These findings provide evidence of crosstalk between p38 MAPK and NF-κB p65 and demonstrate a possible neuroprotective role for the p38 MAPK-NF-κB pathway through Bcl-2 and Bcl-XL in retinal I/R injury in rats.

## Introduction

Nuclear factor kappa-light-chain-enhancer of activated B cells (NF-κB) is an important transcription activator in inflammation, which can activate the expression of genes such as numerous cytokines, cytokine receptors, and adhesion molecules after its nuclear translocation [1]. NF-κB function in inducing or preventing apoptosis depends on different external stimuli. Reactive oxygen species such as superoxide and hydrogen peroxide can cause the pathogenesis of neuronal ischemia/reperfusion (I/R) injury through the NF-κB pathway [2]. Aspirin, an inhibitor of NF-κB activation in hippocampal neurons, is against glutamate-induced cell death in neuron I/R injury [3]. Studies have shown that activating NF-κB can induce apoptosis. However, some researchers have demonstrated that activating NF-κB protects cells from apoptosis [4-7]. In addition, activating NF-κB with tumor necrosis factor α in vivo has been shown to be neuroprotective in neonatal cerebral I/R injury [8]. Therefore, NF-κB activation may have anti- or proapoptotic effects in different types of cells or under different pathological conditions.

Ischemia is a major contributor to retinal injury in various forms of retinopathies. In previous studies, apoptotic cells appeared as early as 3 h after ischemia injury, progressively increased, and peaked at 24 to 48 h [9-11]. The apoptotic cells are mainly located in the inner retina, including the ganglion cell layer (GCL), the inner nuclear layer (INL), and the inner plexiform layer (IPL) [12]. Although, we reported previously that the number of retinal ganglion cells (RGCs) was significantly decreased when using inhibitors of NF-κB p65 after retinal I/R [13], the mechanism of the neuroprotection of NF-κB is still unclear.

B cell lymphoma (Bcl)-2 has been shown to inhibit apoptosis and enhance cell survival under diverse conditions in various cell types. Bcl-X regulates cell death. *Bcl-X* transcripts are spliced into long and short forms, in which the long form (Bcl-XL) suppresses cell death [14,15]. In the nervous system, Bcl-2 has been shown to prevent ischemia-induced neuronal death [16]. Meanwhile, Bcl-2 and Bcl-XL have been expressed in neurons after ischemic brain injury, which protects neuronal cells from apoptosis [17]. NF-κB can induce Bcl-2 or Bcl-XL for survival signaling in neurons and B lymphocytes [18,19]. However, the expression of Bcl-2 and Bcl-XL in retinal I/R injury is unclear.

Recent evidence showed that the p38 mitogen-activated protein kinase (MAPK) and NF-κB pathways are involved in H_2_O_2_-induced interleukin-6 (IL-6) expression in vitro [20]. In our previous study, we demonstrated the NF-κB pathway was involved in IL-6 expression in retinal I/R injury and protected retinal ganglion cells from death [13]. From above, we hypothesize that NF-κB activity induces antiapoptotic Bcl-2 and Bcl-XL and the p38 MAPK-NF-κB pathway plays an important role in protecting RGCs after retinal I/R. In this study, the hypothesis was examined by measuring the expression of p38 MAPK, NF-κB p65, Bcl-2, and Bcl-XL, evaluating the effects of a p38 MAPK inhibitor, and *NF-κB* short interfering RNA (siRNA) on the expression of Bcl-2 and Bcl-XL and cell apoptosis and survival after retinal I/R. Relevant signal pathways were also explored.

## Methods

### Animals

All animal experiments were performed in accordance with the Statement for the Use of Animals from the Association of Research of Vision and Ophthalmology (ARVO). Adult male rats (55–60 days old) from the Military Medical Sciences Animal Center (Beijing, China) were used in this study. The method for inducing transient retinal ischemia was performed according to a previously published study [13]. In brief, after sodium pentobarbital (10 mg/kg bodyweight) was injected, the animals were topically anesthetized with 0.5% tetracaine hydrochloride (Sigma-Aldrich, St. Louis, MO). Then, a 27-gauge needle connected with a saline column was inserted into the anterior chamber through the cornea near the corneoscleral limbus. Intraocular pressure was elevated to 110 mmHg by raising the saline column. Retinal ischemia was confirmed by the whitening of the anterior segment of the eye, blanching of the retinal vessels, and loss of the red reflex with ophthalmoscopic examination. After 60 min of dripping, the needle was gently withdrawn from the anterior chamber, and reperfusion was established. Inhibitor of p38 MAPK (4-(4-fluorophenyl)-2-(4-methylsulfinylphenyl)-5-(4-pyridyl)1H-imidazole; SB203580; Calbiochem, Darmstadt, Germany) and *NF-κB* siRNA (Sangon Biotech, Shanghai, China) were used in this study according to a previous study [13,20]. Rats were euthanized at different times by intraperitoneal injection with lethal doses of sodium pentobarbital (115 mg/kg), and their eyes were enucleated. Rats with cataracts, hemorrhages in the anterior chambers, or endophthalmitis were excluded from this study.

### p38 mitogen-activated protein kinase, nuclear factor kappa-light-chain-enhancer of activated B cell p65, B cell lymphoma-2, and B cell lymphoma-XL expression after retinal ischemia/reperfusion

Retinas at 0 (n=6), 2 (n=6), 4 (n=6), 6 (n=6), 8 (n=6), 12 (n=6), and 24 h (n=6) after retinal I/R were obtained. Normal retinas (n=10) were taken as the control. Total RNA was isolated from retinas using TRIzol Reagent (Invitrogen, Carlsbad, CA) according to the manufacturer’s protocol. DNA contamination was eliminated with a DNase I kit (Ambion, Austin, TX). Reverse transcription was performed using the SuperScript III First-strand Synthesis System (Invitrogen) according to the manufacturer’s instruction. The SYBR Premix Ex Taq real-time PCR kit (Takara, Dalian, China) was used for real-time polymerase chain reactions (PCRs) according to the manufacturer’s instructions. Primers were used as shown in Table 1. All samples were normalized to ribosomal protein L −32 like (*L-32*) and expressed as a fold change. Real-time PCR was performed on ABI 7500 (Applied Biosystems, Foster City, CA). The following conditions were used: predenaturing at 95 °C for 30 s and amplification at 95 °C for 5 s and 58 °C for 34 s, 40 cycles. Six independent eyes were used in each group, and PCR was run in triplicate and repeated twice.

**Table 1:** Primers used for real-time PCR

**Gene**	**Primers (5′-3′)**
*p38MAPK*	F:GAGCTGAAGATTCTGGATTTTGG
	R:TAGCCACGTAGCCGGTCATT
*NF-κBp 65*	F:CTTCTGGGCCATATGTGGAGAT
	R:TCGCACTTGTAACGGAAACG
*Bcl-2*	F:CGACTTTGCAGAGATGTCCA
	R:ATGCCGGTTCAGGTACTCAG
*Bcl-XL*	F:GGTGAGTCGGATTGCAAGTT
	R:GCAGAACTACACCAGCCACA
*L-32*	F:TAAGCGAAACTGGCGGAAAC
	R:CAGGATCTGGCCCTTGAATCT

### Nuclear factor kappa-light-chain-enhancer of activated B cells p65 activity measured after retinal ischemia/reperfusion

At 0 (n=6), 2 (n=6), 4 (n=6), 6 (n=6), 8 (n=6), 12 (n=6), and 24 h (n=6) after retinal I/R injury, the eyes were obtained. Normal eyes (n=10) were used as the control. Retinas were dissected, cut into small pieces, and washed in 0.5 volume of phosphate-buffered salines (PBS) without calcium, magnesium, and phenol red. The tissue samples were placed in a 2 ml microcentrifuge tube, and 500 μl lysis buffer (10 mM Tris-HCl, pH 7.8, 1.5 mM EDTA, 10 mM KCl, 0.5 mM dithiothreitol, 1 mg/ml aprotinin, leupeptin, and pepstatin A, 1 mM 4-(2-aminoethyl) benzenesulfonyl fluoride, 1 mM sodium orthovanadate, 0.5 mM benzamidine, and 2 mM levamisole; Sigma-Aldrich) was added. After being disrupted for 10 s with a Tissue Ruptoe set at the lowest speed, 500 μl lysis buffer was added to the tissue samples and incubated for 15 min on ice. The lysate were transferred to a clean prechilled microcentrifuge tube, and then 10% Nonidet P-40 (W/v; Sigma-Aldrich) was added and centrifuged for 5 min at 10,000× g at 4 °C. After the supernatant was discarded, the pellet was resuspended in 100 μl lysis buffer and recentrifuged. The nuclei pellet was resuspended in 50 μl hypertonic buffer (20 mM Tris-HCl, pH7.8, 150 mM NaCl, 50 mM KCl, 1.5 mM EDTA, 5 mM dithiothreitol, 1 mg/ml aprotinin, leupeptin, and pepstatin A, 1 mM 4-(2-aminoethyl) benzenesulfonyl fluoride, 1 mM sodium orthovanadate, 0.5 mM benzamidine, and 2 mM levamisole; Sigma-Aldrich) and incubated for 30 min at 4 °C. The suspension of nuclei was centrifuged for 10 min at 12,000× g at 4 °C, and then transferred into a new microcentrifuge tube and stored at −80 °C until use.

Equal quantities of total nuclear protein (20 μg) were assayed for NF-κB p65 binding activity by using the Trans-AM NF-kB p65 transcription factor assay kit (Active Motif, Carlsbad, CA) according to the manufacturer’s instructions. NF-κB activity was described as x-fold expression over the control.

### p38 mitogen-activated protein kinases inhibitor treatment and nuclear factor kappa-light-chain-enhancer of activated B cells activity measure

Five mM (2 μl) p38 MAPK inhibitor SB-203580 (in 0.1% dimethyl sulfoxide, dimethyl sulfoxide [DMSO; Sigma-Aldrich]) from Calbiochem was used in I/R injury. The solution of vehicle or SB203580 was infused into the anterior chamber immediately after ischemia was induced. Four groups of rats (six per group) were employed to study the effect of p38 MAPK inhibition on nuclear NF*-*κB activity. Normal retinas were taken as control. The other three groups were I/R, I/R+vehicle (0.1% DMSO), and I/R+SB203580. The eyes were enucleated at 8 h after I/R. Retinal nuclear proteins extraction and NF-κB activity measure were performed as above. NF-κB activity was described as × fold expression over the control.

### B cell lymphoma-2 and B cell lymphoma-XL expression after p38 mitogen-activated protein kinases inhibitor treatment

The animals treated with SB-203580 as above were euthanized at 8 h after I/R and their eyes enucleated. Retinal mRNAs were extracted from four groups (normal retina, I/R, I/R+ vehicle [0.1% DMSO], and I/R+SB203580), and the mRNA levels of *Bcl-2* and *Bcl-XL* were measured with real-time PCR.

### B cell lymphoma-2 and B cell lymphoma-XL expression after treatment with nuclear factor kappa-light-chain-enhancer of activated B cells p65 short interfering RNA

The 21-nucleotide-long siRNAs of *NF-κB* and the control were the same as our previous study [13]. A non-specific, irrelevant siRNA duplex with random nucleotides and a GC ratio close to that of siRNA against NF-κB p65 was used as control. The sequences of NF-κB p65 siRNA and control siRNA duplex were shown in Table 2. Five μg siRNA was immediately injected into the vitreous cavity after ischemia was induced. At 8 h after reperfusion, the animals were euthanized and their eyes enucleated. Retinal mRNAs were extracted, and the mRNA levels of *Bcl-2* and *Bcl-XL* were measured with real-time PCR. Four groups of rats (six per group) were employed to study the effect of inhibiting NF*-*κB activity. Normal retinas were taken as the control. The other three groups were I/R, I/R+control of siRNA in vehicle (Lipofectamine 2000 [Invitrogen] and Dulbecco's Modified Eagle Medium [DMEM]/F12: no serum and no antibiotics), and I/R+siRNA.

**Table 2:** Sequence of siRNAs

**Gene**	**Sequence (5′-3′)**
*NF-κB p65*	F: UACGCGAGCAACAGGAACAUU
	R: UGUUCCUGUUGCUCGCGUAUU
*Control siRNA duplex*	F: AUUGUAUGCGAUCGCAGACUU
	R: PGUCUGCGAUCGCAUACAAUUU

### Assessment of apoptosis

Six groups (six rats in each group) were set up to detect the effects of the p38 MAPK inhibitor and *NF-κB p65* siRNA on retinal cell apoptosis. Normal retinas were the control group. The other five groups were individually I/R, I/R+vehicle (0.1% DMSO), I/R+control of siRNA in vehicle (Lipofectamine 2000 and DMEM/F12: no serum and no antibiotics), I/R+SB203580, and I/R+*NF-κB* siRNA. The presence of nucleosomes from apoptosis can be quantified by using a Cell Death Detection enzyme-linked immunosorbent assay (ELISA; Roche Applied Science, Indianapolis, IN) with antibodies directed against DNA and histones. Briefly, 48 h after retinal I/R, animals were euthanized by intraperitoneal injection with lethal doses of sodium pentobarbital (115 mg/kg), and the retinas were removed. These retinas were homogenized in 400 μl lysis buffer using a homogenizer and incubated for 30 min at room temperature. After spinning at 200 g for 10 min, the supernatant was further diluted 1:200 in lysis buffer. Twenty μl final dilution was used to detect apoptosis according to the manufacturer’s protocol.

### Cell survival in the ganglion cell layer

Six groups (six rats in each group) as above were set up to detect the effects of the p38 MAPK inhibitor and *NF-κB p65* siRNA on retinal cell survival. Rats were euthanized by intraperitoneal injection with lethal doses of sodium pentobarbital (115 mg/kg) at 7 days after retinal I/R. The eyes were enucleated and fixed in 4% paraformaldehyde (Sigma-Aldrich) for 2 h at room temperature. The whole mounted retinas and subsequent cresyl violet stained cells count in the RGC layer were performed according to previously described methods [13,21]. In brief, after the retinas were dissected, they were mounted flatly on a slide with vitreous side upward and dried overnight. Then, the specimens were stained with 1% cresyl violet. After dehydration, the retinas were covered with coverslips. This method is reliable for studying quantitative changes of RGCs, which has been used in many published papers to show the viable RGCs, by avoiding damage with retrograde labeling. Cresyl violet stained RGCs were counted in the central retina and the peripheral retina.

### Statistical analysis

Real-time data analysis was performed with relative quantification using the 2^-ΔΔCT^ method according to Livak and Schmittgen [22]. Gene expression was normalized by gene expression of L-32 and then described as the × fold expression over the control.

Data were given as mean±standard deviation (SD) and statistically analyzed using ANOVA (ANOVA) and the post hoc *t* test method. Statistical significance was set at p<0.05.

## Results

### mRNA expression of nuclear factor kappa-light-chain-enhancer of activated B cells p65, p38 mitogen-activated protein kinases, B cell lymphoma 2, and B cell lymphoma-XL in the retina after ischemia/reperfusion

**Figure 1:**
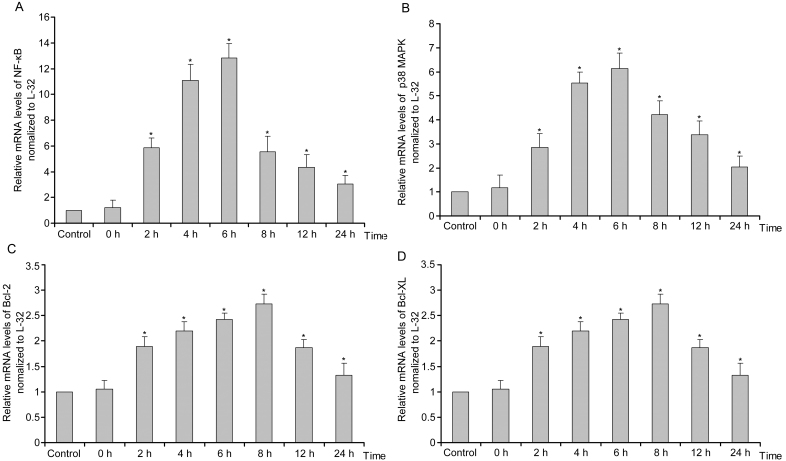
mRNA expression of p38 mitogen-activated protein kinases (*MAPK*), nuclear factor kappa-light-chain-enhancer of activated B cells (*NF-κB*) *p65*, B cell lymphoma (*Bcl*)*-2*, and *Bcl-XL* in retinal ischemia/reperfusion (I/R) injury. **A**: Relative *p38 MAPK* mRNA levels significantly increased, peaked at 6 h, and then gradually decreased. **B**: Relative *NF-κB* mRNA levels significantly increased, peaked at 6 h, and then gradually decreased. **C**, **D**: Relative *Bcl-2* and *Bcl-XL* mRNA levels initially decreased, increased to the peak at 8 h, and then gradually decreased. *Statistically significant compared to the control: p<0.05. *L-32* was the housekeeping gene. n=10 for Control and n=6 for other groups. mRNA expression level is expressed as mean±standard deviation (SD).

*NF-κB p65* mRNA levels showed a time-dependent change after retinal I/R (Figure 1A). *NF-κB p65* mRNA expression increased rapidly at 2 h (p<0.05) and peaked at 6 h (p<0.05). Then the *NF-κB p65* level decreased gradually. The mRNA expression of *p38 MAPK* increased and reached a peak at 6 h (p<0.05). Afterward, the level of *p38 MAPK* decreased gradually (Figure 1B). The mRNA levels of *Bcl-2* and *Bcl-XL* significantly increased at 2, 4, and 6 h, and peaked at 8 h (p<0.05). Afterward, the mRNA expression of *Bcl-2* and *Bcl-XL* decreased, but remained at a higher level compared with the control (Figure 1C,D).

### Activity of nuclear factor kappa-light-chain-enhancer of activated B cells p65 in nucleus after ischemia/reperfusion

**Figure 2:**
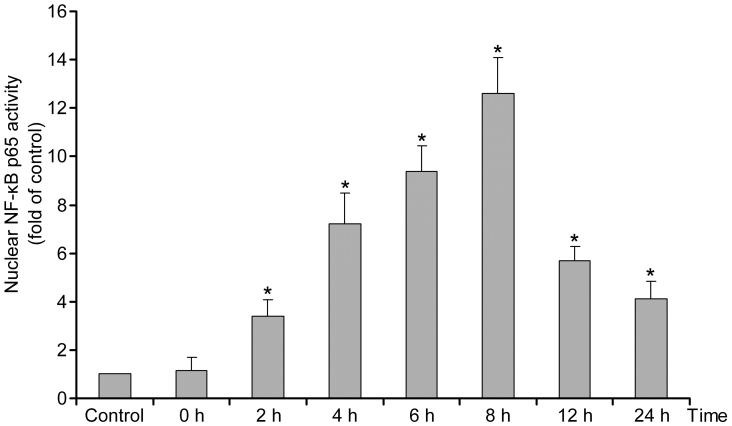
Nuclear factor kappa-light-chain-enhancer of activated B cells (NF-κB) p65 nuclear activity via ELISA in retinal ischemia/reperfusion (I/R) injury. NF-κB p65 nuclear translocation gradually increased at 2 h, 4 h, and 8 h. Afterward, the level of NF-κB decreased but remained at a higher level than the control. *Statistically significant compared to the control: p<0.05. n=10 for Control and n=6 for other groups. Nuclear NF-κB p65 activity were given as mean±standard deviation (SD).

Because NF-κB p65 regulates the expression of genes in the nucleus, we detected whether NF-κB p65 was located in the nucleus after retinal I/R via ELISA. In the control group, the level of NF-κB p65 in the nucleus was low, which indicated that NF-κB p65 is mainly located in cytoplasm. At 2, 4, 6, 8, 12, and 24 h after retinal I/R, NF-κB p65 was located in the nucleus at a higher concentration compared with the control (p<0.05). The nuclear concentration of NF-κB p65 decreased gradually after 8 h (Figure 2) but remained higher than that of the control.

### Effect of p38 mitogen-activated protein kinases inhibitor on nuclear factor kappa-light-chain-enhancer of activated B cells p65 activity

**Figure 3:**
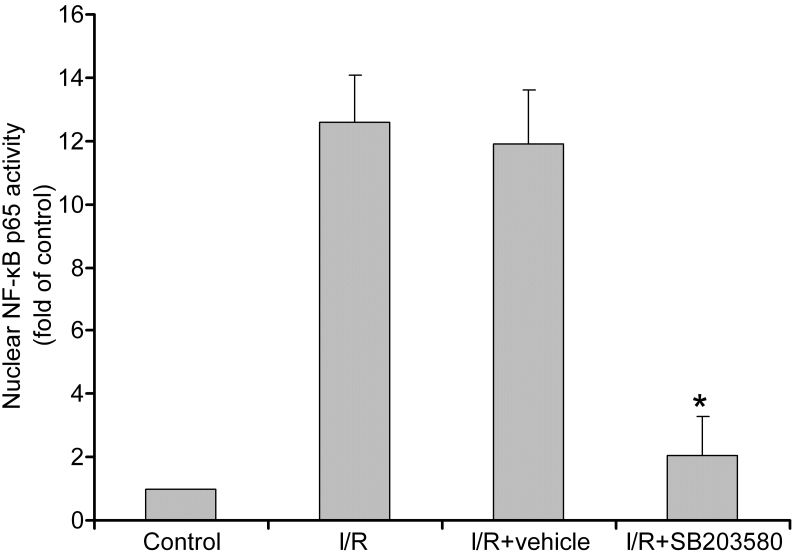
Effect of p38 mitogen-activated protein kinases (MAPK) inhibitor on nuclear factor kappa-light-chain-enhancer of activated B cells (NF-κB) activity. After application of p38 MAPK inhibitor SB203580, the activity of nuclear NF-κB obviously decreased when compared to the ischemia/reperfusion (I/R) control and vehicle groups at 8 h after ischemia/reperfusion (I/R). *Statistically significant compared to the I/R control: p<0.05. n=6 in each group. Nuclear NF-κB p65 activity were given as mean±standard deviation (SD).

After treatment with p38 MAPK inhibitor SB203580, nuclear NF-κB p65 activity decreased significantly compared with the non-inhibitor treated control (p<0.05) and showed no difference compared with the normal control (p>0.05; Figure 3). These indicated that the increase in NF-κB p65 levels in the nucleus induced with I/R was completely abolished by the p38 MAPK inhibitor.

### Expression of B cell lymphoma-2 and B cell lymphoma-XL in retinas after treatment with p38 mitogen-activated protein kinases inhibitor and nuclear factor kappa-light-chain-enhancer of activated B cells p65 short interfering RNA

**Figure 4:**
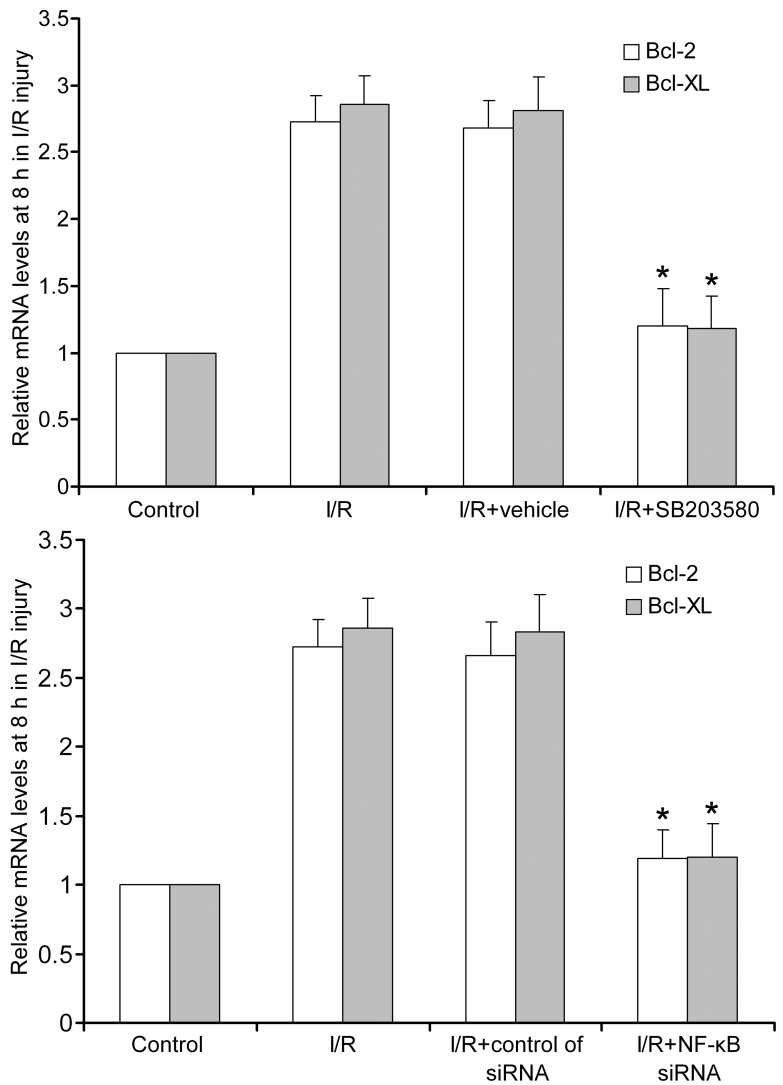
B cell lymphoma (*Bcl*)*-2* and *Bcl-XL* mRNA expression after treatment of p38 mitogen-activated protein kinases (MAPK) inhibitor and nuclear factor kappa-light-chain-enhancer of activated B cells (*NF-κB*) siRNA. SB203580 and *NF-κB* siRNA inhibited *Bcl-2* and *Bcl-XL* mRNA expression at 8 h after ischemia/reperfusion (I/R). *Statistically significant compared to the I/R control: p<0.05. n=6 in each group. *Bcl-2* and *Bcl-XL* mRNA expressions were given as mean±standard deviation (SD)

With p38 MAPK inhibitor SB203580 or *NF-κB p65* siRNA, the mRNA transcripts of *Bcl-2* and *Bcl-XL* had no significant difference compared with the normal group (p>0.05) but obviously decreased compared with the I/R group (p<0.05; Figure 4). These results indicated that the expression of *Bcl-2* and *Bcl-XL* could be prevented with the p38 MAPK inhibitor and *NF-κB p65* siRNA.

### Increases in retinal cells apoptosis by p38 mitogen-activated protein kinases inhibitor and nuclear factor kappa-light-chain-enhancer of activated B cells p65 short interfering RNA

**Figure 5:**
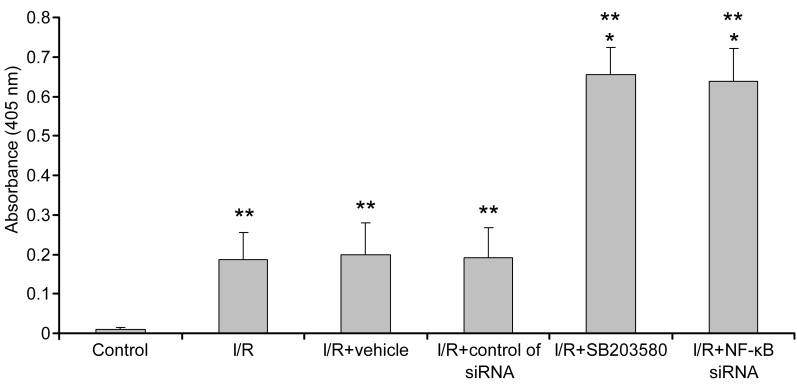
Effects of p38 mitogen-activated protein kinases (MAPK) inhibitor and nuclear factor kappa-light-chain-enhancer of activated B cells (*NF-κB*) siRNA on nucleosome release. The apoptosis of retinal tissues was evaluated by nucleosome release ELISA. Eyes injected with SB203580 or *NF-κB p65* siRNA had higher nucleosome release than the ischemia/reperfusion (I/R) control groups. Control: normal retinas, the other five groups were individually as I/R, I/R+vehicle (0.1% DMSO), I/R+control of siRNA in vehicle (Lipofectamine 2000 [Invitrogen] and DMEM/F12: no serum and no antibiotics), I/R+SB203580 and I/R+*NF-κB* siRNA. *Statistically significant compared to the I/R control: p<0.05. ** Statistically significant compared to the normal control: p<0.05. n=6 in each group. Cell number was given as mean±standard deviation (SD).

The apoptosis of retinal tissues was evaluated with nucleosome release ELISA. The nucleosome release assay evaluated cell apoptosis throughout the retina. Higher absorbance is correlated with increased apoptosis due to detection of histone-associated DNA fragments. Eyes after the retinal I/R injury showed obviously higher absorbance than the normal control (p<0.05), and the eyes injected with SB203580 or *NF-κB p65* siRNA had higher nucleosome release than the I/R control groups (p<0.05; Figure 5). These data indicated that inhibiting the p38 MAPK-NF-κB pathway increased retinal cell apoptosis.

### Decreases in the number of survival cells in the retinal ganglion cell layer by p38 mitogen-activated protein kinase inhibitor and nuclear factor kappa-light-chain-enhancer of activated B cells p65 short interfering RNA

**Figure 6:**
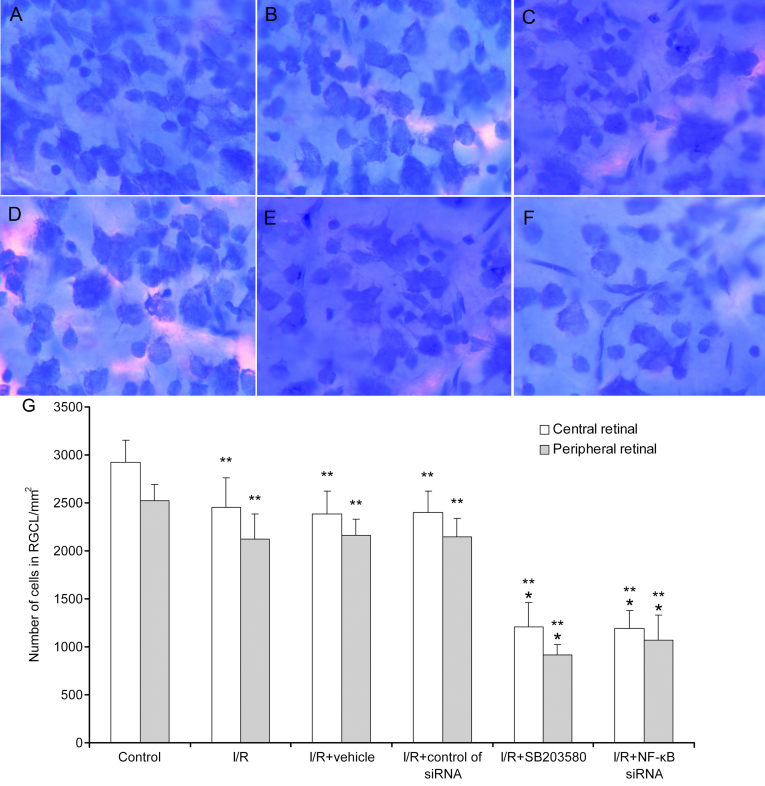
Effects of p38 mitogen-activated protein kinases (MAPK) inhibitor and nuclear factor kappa-light-chain-enhancer of activated B cells (*NF-κB*) siRNA on retinal ganglion cells (RGCs) survival in retinal ischemia/reperfusion (I/R) injury. Control: normal retinas (**A**), the other five groups were individually as I/R (**B**), I/R+vehicle (0.1% DMSO; (**C**), I/R+control of siRNA in vehicle (Lipofectamine 2000 [Invitrogen] and DMEM/F12: no serum and no antibiotics; **D**) I/R+SB203580 (**E**) and I/R+*NF-κB* siRNA (**F**). The number of cells in the RGCL significantly was decreased in the central and peripheral retina by SB203580 and *NF-κB* siRNA when compared to the normal and I/R control (**G**). *Statistically significant compared to the I/R group: p<0.05. ** Statistically significant compared to the normal control: p<0.05. Data were given as mean±standard deviation (SD).

To evaluate the effects of p38 MAPK and NF-κB p65 on cell survival in the RGC layer after retinal I/R, we stained the RGCs with cresyl violet and counted RGCs at 7 days after using p38 MAPK inhibitor SB203580 and *NF-κB p65* siRNA (Figure 6A-F). The number of surviving cells showed a significant decrease (p<0.05) in the central and peripheral retina after treatment with SB-203580 and *NF-κB p65* siRNA compared with I/R retinas (Figure 6G). In addition, the surviving cells in the I/R groups decreased significantly compared with the normal group (p<0.05).

## Discussion

In this study, we demonstrate that the p38 MAPK-NF-κB p65 signaling pathway is induced to regulate Bcl-2 and Bcl-XL expression, apoptosis, and survival of RCGs in retinal I/R injury. We provide evidence indicating that p38 MAPK and NF-κB P65 are elements of the neuroprotection cascade: 1) p38 MAPK was activated accompanied by the activation of NF-κB p65 in retinal I/R injury; 2) NF-κB p65 activation was blocked by pretreatment with the p38 MAPK inhibitor; 3) Bcl-2 and Bcl-XL expression was blocked by the p38 MAPK inhibitor and *NF-κB p65* siRNA; and 4) nucleosome release increased and cell survival decreased after using the p38 MAPK inhibitor and *NF-κB p65* siRNA. Therefore, we propose a signaling pathway by which NF-κB p65 acts as a downstream effecter of p38 MAPK to protect retinal cells from apoptosis through Bcl-2 and Bcl-XL in retinal I/R injury.

Activation of the p38 MAPK-NF-κB signaling pathway has been shown in different stimuli in different cell systems. A previous report demonstrated that the p38 MAPK and NF-κB signaling pathways are involved in high glucose-induced inflammation in human monocytes, and p38 MAPK acts as an upstream factor leading to NF-κB activation [23]. Meanwhile, recent evidence showed that the p38 MAPK-NF-κB signaling pathway is involved in H_2_O_2_-induced IL-6 expression by retinal pigment epithelial cells in vitro. p38 MAPK and NF-κB expression increased after high glucose and H_2_O_2_ treatment, and the p38 MAPK inhibitor abolished the increase of NF-κB in nuclear extracts [20]. From our data, we demonstrated that activation of NF-κB depends on functional p38 MAPK in retinal I/R injury, which is consistent with these studies [20,23].

Proapoptotic and antiapoptotic roles of NF-κB in neuronal and glial cells have been identified in several central nervous system neurodegenerative diseases [13,24-32]. A study showed that activation of NF-κB may be associated with photoreceptor cell death through upregulation of gene expression of proinflammatory and neurotoxic molecules (e.g., tumor necrosis factor α) in microglial cells in the retinal degeneration (rd) retina [25]. Meanwhile, some studies have elucidated that NF-κB promotes apoptosis in certain cells such as neurons and Schwann cells [26,27]. Chen et al. [28] thought activation of NF-κB p65 appeared to play an important role in mouse retinal degeneration following retinal I/R injury. However, many other studies have demonstrated that NF-κB is involved in antiapoptosis [13,24,29-32]. The decrease of NF-κB in cultured 661W mouse photoreceptor cells resulted in light-induced photoreceptor apoptosis [24]. In addition, nitrosative stress induced apoptosis through inhibiting NF-κB [32]. In our previous research, we demonstrated the inhibition of NF-κB p65 decreased retinal ganglion cell survival in retinal I/R injury [13]. In this study, *NF-κB p65* siRNA blocked the NF-κB signaling pathway, which led to inhibited Bcl-2 and Bcl-XL expression, increased nucleosome release, and decreased survival of RGCs in retinal I/R injury. Thus, we further confirmed that NF-κB plays an important role in neuroprotection through regulating the expression of Bcl-2 and Bcl-XL in retinal I/R injury. Endogenous early-activated NF-κB upregulated the expression of Bcl-2 and Bcl-XL, which is consistent with a previous study [31]. However, although *Bcl-2* and *Bcl-XL* mRNA expression increased in the retinal I/R group, cell death still occurred after retinal I/R compared with the normal group. These observations suggest that neuroprotection in the normal group might be through other pathways in addition to the NF-κB pathway, which requires further studies. Increases of Bcl-2 and Bcl-XL induced by early-activated NF-κB could partly protect retinal cells from apoptosis.

In this study, the p38 MAPK inhibitor prevented NF-κB p65 activity, which implied p38 MAPK has a function in regulating neuroprotection. We observed that *p38 MAPK* mRNA expression increased, reached a peak at 6 h, and remained elevated after transient ischemia, which was followed by the Bcl-2 and Bcl-XL increase. Thus, we further observed Bcl-2 and Bcl-XL expression after application of the p38 MAPK inhibitor. The data demonstrated that p38 MAPK can control Bcl-2 and Bcl-XL expression. According to other studies [6,7,31] and our data, NF-κB families activate the expression of Bcl-2 and Bcl-XL. Thus, we guess that the effect of p38 MAPK on Bcl-2 and Bcl-XL is indirectly through the NF-κB signaling pathway, which must be further explored.

In previous studies, inhibition of p38 MAPK significantly decreased apoptosis and promoted recovery in rats [23,33,34]. p38 MAPK inhibitor SB203580 inhibited light-induced apoptosis in photoreceptor cells [23], increased the number of surviving RGCs in a dose-dependent manner in axotomy-induced apoptosis of RGCs [33], and played a neuroprotective role in forebrain global ischemia [34]. However, in our data, p38 MAPK activated antiapoptosis genes Bcl-2 and Bcl-XL, and promoted cell survival, which could explain that p38 MAPK protects retinal cells from ischemia injury. Our results are consistent with a prior ischemic preconditioning (IPC) study [35]. We elevated transiently the intraocular pressure (IOP) to 110 mmHg for 1 h and then established reperfusion in rats. For IPC, IOP was increased to 160 mmHg for 8 min, followed by 110 mmHg IOP for 45 min after 24 h. Before IPC, *p38α* siRNA or p38 MARK inhibitor SB203580 treatment significantly attenuated the neuroprotective effect of IPC [35]. In our data, p38 MARK inhibitor SB203580 applied immediately after ischemia was introduced increased retinal cell apoptosis, decreased the number of survival RGCs, and blocked Bcl-2 and Bcl-XL expression. In these two studies, early endogenous p38 MAPK was inhibited, resulting in retinal cell apoptosis, which indicated that early transient p38 MAPK expression has a neuroprotective effect. However, Roth et al. [36] reported that blocking activation of p38 MAPK significantly decreased thinning of the inner nuclear layers’ thickness, and decreased the percentage of terminal deoxynucleotidyl transferase dUTP nick end labeling-positive cells. As Roth et al. [36] mentioned, SB203850 was injected into the vitreous 15 min before and immediately after ischemia was completed, which inhibited not only early p38 MAPK activity but also its late function. However, only early p38 MARK activity was blocked in our research, which is consistent with the research of Dreixler et al. [35]. Thus, although p38 activation contributes to I/R injury [36], early activation plays an important role in subsequent protection from ischemia. Meanwhile, in Roth’s studies [35,36] SB203580 appeared to block p38 specifically and not other MAPKs, c-Jun N-terminal kinase, or extracellular signal-regulated kinase; however, Muniyappa et al. [37] demonstrated that SB203580 can induce the activation of the c-Jun N-terminal kinase pathway. Other signaling pathways besides p38/NF-κB may be involved in retinal I/R injury, which needs to be further explored.

In summary, endogenous NF-κB appears to play a critical role in neuroprotection of retinal cells in retinal I/R injury, which may be through controlling Bcl-2 and Bcl-XL expression. Meanwhile, endogenous early-activated p38 MAPK may be involved in retinal protection in retinal I/R injury through regulating the NF-κB pathway.
